# Clinical characteristics and management of primary retroperitoneal sarcoma: A literature review

**DOI:** 10.1002/ags3.12756

**Published:** 2023-11-16

**Authors:** Yukinori Yamagata, Motokiyo Komiyama, Shintaro Iwata

**Affiliations:** ^1^ Department of Gastric Surgery National Cancer Center Hospital Cyuo‐ku Japan; ^2^ Department of Urology National Cancer Center Hospital Cyuo‐ku Japan; ^3^ Department of Musculoskeletal Oncology and Rehabilitation National Cancer Center Hospital Cyuo‐ku Japan

**Keywords:** resection, retroperitoneal sarcoma, treatment

## Abstract

Retroperitoneal sarcoma (RPS) is a rare tumor classified into many histological types. It is also often detected only after it has grown to a considerable size and requires extensive resection of the surrounding organs, making it difficult to offer optimal patient‐tailored management. Evidence supporting specific treatment modalities for RPS is insufficient, owing to its rarity. The Japanese clinical practice guidelines for RPS were published in December 2021, with the aim of accumulating existing evidence and indicating the optimal practice for RPS. These guidelines provide important clinical questions (CQs) concerning the diagnosis and treatment of RPS. This review, with a particular focus on primary RPS, attempts to introduce clinical problems in the diagnosis and treatment of RPS and to assess those problems along with the CQs in the guidelines. According to these guidelines, although chemotherapy and radiotherapy are expected to have therapeutic effects, the level of evidence to support these treatments is not very high at present. Accordingly, complete resection of the tumor is the first and only option for managing primary RPS. However, as with other tumors, the demand for multidisciplinary treatment for RPS is increasing. These guidelines will undoubtedly represent a milestone in clinical practice in relation to RPS in the future, and further evidence is expected to be accumulated based on the CQs that have been proposed.

## Graphical abstract

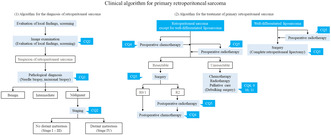

## 1 INTRODUCTION

Retroperitoneal sarcoma (RPS) is a rare tumor that accounts for 0.15% of all malignant tumors and approximately 15% of all sarcomas.^1^ However, RPSs can be classified into many histological types, such as liposarcoma, leiomyosarcoma, undifferentiated pleomorphic sarcoma, and others. Therefore, it is difficult to offer optimal patient‐tailored management.

Owing to its rarity, there is a strong demand for clinical guidelines for RPS to assist in daily clinical practice. In Western countries, clinical guidelines for RPS have been established by the Trans‐Atlantic Retroperitoneal Sarcoma Working Group.^2^ However, only guidelines for soft tissue tumors, which include but are not specific for RPS, have been established in Japan, by the Japanese Orthopaedic Association (JOA).^3^

As mentioned above, RPS accounts for only 15% of all sarcomas, and the histology of RPS differs from that of other sarcomas.^4^ RPS is rarely found when the tumor is small, as symptoms are less common in the early stages than in later ones.^4^ Thus, the tumor is typically found after it has grown to a considerable size and often requires extensive resection of the surrounding organs. For this reason, the prognosis of RPS is worse than that of other sarcomas.^3^

For advanced cases of other cancers, multidisciplinary treatment combining surgery with chemotherapy and/or radiotherapy is becoming the mainstream approach, and it is presumed that this trend is the same for RPS. Since RPS is a rare disease, however, evidence is scarce. Therefore, the accumulation of existing evidence is an urgent task. The Japanese clinical practice guidelines for retroperitoneal sarcoma were published in December 2021.^4^

In this article, we will examine the clinical features by reviewing the previous literature, introducing new guidelines and comparing them with Japanese soft tissue tumor guidelines, and proposing appropriate treatments for RPS.

## 2 CLINICAL CHARACTERISTICS OF RPS

### 2.1 Clinicopathological grading

The pathological diagnosis of sarcoma is difficult for several reasons, as follows: (1) the histological diagnosis of sarcoma itself has changed significantly with time (e.g., malignant fibrous histiocytoma was renamed undifferentiated pleomorphic sarcoma in the 4^th^ edition of the WHO classification in 2013, and 13 diseases not included in the 4^th^ edition were added to the 5^th^ edition in 2020); (2) approximately 5% of sarcomas are unclassifiable; and (3) with the introduction of genetic testing, many tumors with new fusion genes have been identified, and tumors that were previously thought to be the same tumor are now known to follow different clinical courses (e.g., Ewing sarcoma and CIC sarcoma).^5^, ^6^, ^7^, ^8^ Retroperitoneal tumors are pathologically categorized as mesodermal, neurogenic, and extragonadal germ cell tumors, all of which include both benign and malignant tumors (Table 1).^9^ More than 90% of retroperitoneal tumors are of mesodermal origin, mostly liposarcoma, leiomyosarcoma, and undifferentiated pleomorphic sarcoma.^10^ However, as shown in Table 1, although the frequency of occurrence is very low, there are many histological types of RPS. The characteristics of each tumor are different, which makes it difficult to determine the optimal treatment strategy for RPS.^11^

**TABLE 1:** Classification of retroperitoneal tumors based on the type of tissue of origin (partially modified).^9^

Tissue of origin	Benign	Malignant
Mesodermal		
Connective tissue	Fibroma	Fibrosarcoma
Fat	Lipoma	Liposarcoma
Smooth muscle	Leiomyoma	Leiomyosarcoma
Striated muscle	Rhabdomyoma	Rhabdomyosarcoma
Blood vessels	Hemangioma	Angiosarcoma
	Hemangiopericytoma	
Lymph vessels	Lymphangioma	Lymphangiosarcoma
Perivascular epithelioid cell	Angiomyolipoma	Sarcoma of perivascular cells
	Lymphangioleiomyomatosis	
	Clear cell “sugar” tumor	
	Clear cell myomelanocytic tumor	
	Pigmented melanotic tumor	
Neurogenic		
Nerve Sheath	Schwannoma, neurofibroma	Malignant schwannoma
		Neurogenic sarcoma
		Neurofibrosarcoma
Sympathetic ganglia	Ganglioneuroma	Malignant paraganglioma or pheochromocytoma
	Ganglioneuroblastoma	
Parasympathetic ganglia	Paraganglioma	Malignant paraganglioma or pheochromocytoma
	Pheochromocytoma	
Extragonadal germ cell		
	Mature teratoma	Seminoma
	Immature teratoma	Malignant teratoma
		Embryonal sac tumor
		Yolk sac tumor
		Choriocarcinoma
Unknown		
		Undifferentiated pleomorphic sarcoma

The French Federation of Cancer Centers (FNCLCC) system is the standard for grading histological malignancies of retroperitoneal tumors.^12^ In this system, tumor differentiation, mitotic count, and tumor necrosis are scored, and the histological grade of the tumor is evaluated in three stages (Table 2). The evaluation of tumor malignancy according to the FNCLCC system is used for tumors in the TNM classification of the Union for International Cancer Control (UICC) and American Joint Committee on Cancer (AJCC).^13^, ^14^ However, in the latest UICC/AJCC TNM 8th edition, the distinction between G2 and G3 is no longer reflected in the tumor staging (Table 3). As mentioned above, the histological diagnosis of sarcoma has evolved significantly since the FNCLCC classification was first proposed in 1996. Furthermore, the prognosis of sarcomas depends more strongly on the histological type than the mitotic status and necrosis.^15^ Moreover, advances in genetic searches have led to advances in the diagnosis of sarcomas. However, the use of the FNCLCC classification is controversial, and a new system for grading the histological malignancy of RPS is required.

**TABLE 2:** The French Federation of Cancer Centers grading system.^12^

Tumor differentiation
Score 1: Sarcomas closely resembling normal adult mesenchymal tissue
Score 2: Sarcomas for which the histological typing is certain
Score 3: Embryonal sarcomas, undifferentiated sarcomas, and uncertain type
Mitotic count
Score 1: 0–9/10HPF
Score 2: 10–19/10HPF
Score 3: ≥20/10HPF
Tumor necrosis
Score 1: No Necrosis
Score 2: ≤50% tumor necrosis
Score 3: >50% tumor necrosis
Histologic grade
Grade 1: Total score 2, 3
Grade 2: Total score 4, 5
Grade 3: Total score 6, 7, 8

Abbreviation: HPF, high‐power field (×400).

**TABLE 3:** UICC/AJCC TNM clinical classification (8th edition).^13^, ^14^

a. T‐primary tumor	
Thoracic and abdominal viscera	
T1	Tumor confined to a single organ
T2a	Tumor invades serosa or visceral peritoneum
T2b	Tumor with microscopic extension beyond the serosa
T3	Tumor invades another organ or macroscopic extension beyond the serosa
T4a	Multifocal tumor involving no more than two sites in one organ
T4b	Multifocal tumor involving more than two sites but not more than five sites
T4c	Multifocal tumor involving more than five sites

b. Stage				
Stage IA	T1	N0	M0	G1, GX low grade
Stage IB	T2, T3, T4	N0	M0	G1, GX Low Grade
Stage II	T1	N0	M0	G2, G3 High Grade
Stage IIIA	T2	N0	M0	G2, G3 High Grade
Stage IIIB	T3, T4	N0	M0	G2, G3 High Grade
	Any T	N1^a^	M0	Any G
Stage IV	Any T	Any N	M1	Any G

Abbreviations: AJCC, American Joint Committee on Cancer; UICC, Union for International Cancer Control.

^a^ AJCC classifies N1 as stage IV for the extremities and superficial trunk.

The TNM classification is also used for the clinical grading of sarcomas. In the current 8th edition of the UICC/AJCC TNM classification, the T category of RPS is evaluated separately from other sarcomas, but the staging is the same as that of other sarcomas (Table 3).^13^, ^14^ RPS has a worse prognosis and greater histologic bias than other sarcomas (liposarcoma and leiomyosarcoma are common in RPS).^16^ Therefore, it is necessary to consider whether or not the staging system used for other sarcomas can also be used for RPS.

### 2.2 Epidemiology

According to the Surveillance Epidemiology and End Results (SEER) database, malignant sarcoma accounts for 1.5% of all malignancies.^17^ Approximately 15% of these are of retroperitoneal origin; hence, RPS is a very rare disease.^18^ Slightly more common in males, two‐thirds of all cases are high‐grade, and approximately 10% metastasize, with the most common sites being the liver and lungs.^18^ As mentioned in the previous section, liposarcoma is most common in adults, followed by leiomyosarcoma and undifferentiated pleomorphic sarcoma.

RPS is often asymptomatic and presumed to have already grown to some extent by the time of its discovery. We attempted to find studies that focused on the size of RPSs. However, we found only one retrospective study that focused on this aspect.^19^ In that study, the authors defined retroperitoneal tumors ≥25 cm in diameter as “giant.” They revealed that the larger the tumor, the higher the proportion of male patients, the greater the proportion of liposarcoma, the fewer symptoms unrelated to the mass, and the lower the R0 resection rate. They also performed univariate and multivariate analyses for some factors and showed that “lesions other than liposarcoma,” “R0 not able to be achieved,” and “stage III tumor” were risk factors associated with a poor prognosis in patients with RPS.

## 3 MANAGEMENT OF PRIMARY RPS

### 3.1 New Japanese clinical practice guidelines for RPS

**FIGURE 1:**
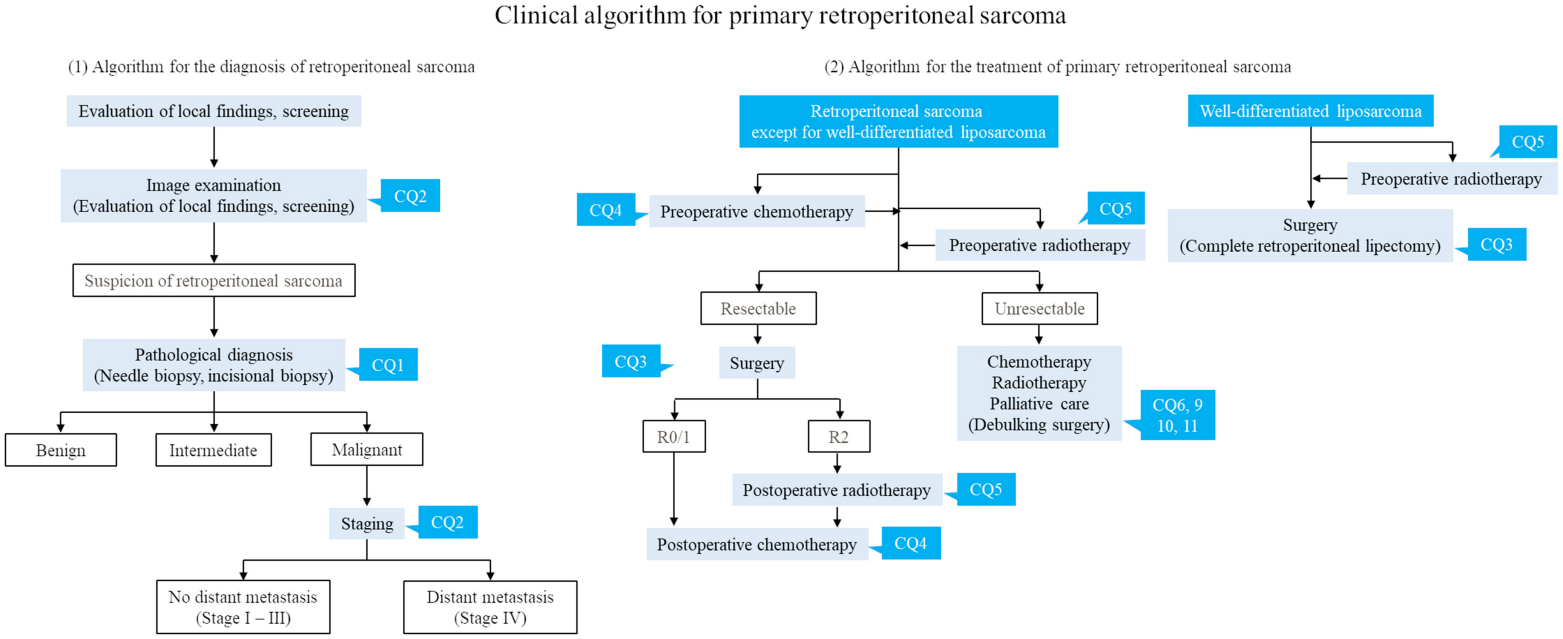
Clinical algorithm for primary retroperitoneal sarcoma.^4^ CQ, clinical question.

In December 2021, independent of the JOA clinical practice guidelines for the management of soft tissue tumors, clinical practice guidelines for the management of retroperitoneal sarcomas were published in Japan.^4^ The guidelines presented a clinical practice strategy for RPS and 11 clinical questions (CQs) based on current clinical issues. CQs related to the diagnosis and treatment of primary RPS were extracted and are presented in Table 4, and treatment strategies for primary RPS are shown in Figure 1.^3^, ^4^ The comparison between the new guidelines of RPS and guidelines for soft tissue tumors is also shown in Table 4.^3^, ^4^ Unfortunately, the level of evidence for all CQs is low, and most items do not provide clear recommendations. However, with the publication of the RPS guidelines, the characteristics and problems of RPS treatment have been clarified. Below, we discuss the clinical issues highlighted in the RPS guidelines and reference the outline of the diagnosis of and management practice for primary RPS by comparing these guidelines with those for soft tissue tumors, showing the difference between them.

**TABLE 4:** CQs presented in the clinical practice guidelines on the management of primary retroperitoneal sarcomas (excerpted and partially modified), and comparison with the JOA clinical practice guidelines on the management of soft tissue tumors.^3^, ^4^

	Important clinical issue	Recommendation (strength of evidence, degree of recommendation^a^)	Description in the JOA clinical practice guidelines on the management of soft tissue tumors (strength of evidence, degree of recommendation^a^)
1. The diagnosis of retroperitoneal tumor			
CQ1	Is a biopsy recommended for diagnosis of retroperitoneal tumors?	Conditionally recommend biopsy in the diagnosis of retroperitoneal tumors (C, 2)	Should not perform unplanned excision (C, 2) Conditionally recommend genetic test in pathological examination of soft tissue tumors (D, 2) Should perform biopsy for the tumor over 5 cm (D, 2) Conditionally recommended excisional biopsy for the tumor less than 2 cm (D, 2)
CQ2	Are MRI and PET/CT recommended for the diagnosis of retroperitoneal tumors?	Should perform MRI and/or PET/CT for the diagnosis of retroperitoneal tumors (C, 2)	Should perform PET/CT before and after treatment (C, 2) Not mentioned about MRI
2. Treatment of primary retroperitoneal sarcoma			
CQ3	Is R0 resection recommended for retroperitoneal sarcoma?	Conditionally recommend R0 resection to retroperitoneal sarcoma (B, 2)	Should perform wide resection for malignant soft tissue tumors (C, 2) Should perform marginal excision for atypical lipomatous tumors (C, 2) Should set surgical margin which take into account the extent of invasion for malignant soft tissue tumors with findings of invasion on imaging study (C, 2)
CQ4	Is adjuvant chemotherapy recommended for primary retroperitoneal sarcoma?	No clear recommendations can be made at this time for adjuvant chemotherapy for primary retroperitoneal sarcoma (D, −)	Conditionally recommend perioperative adjuvant chemotherapy for resectable highly malignant soft tissue tumors (B, 2)
CQ5	Is adjuvant radiotherapy recommended for primary retroperitoneal sarcoma?	No clear recommendations can be made at this time for adjuvant radiotherapy for general primary retroperitoneal sarcoma (D, 2) Suggest adjuvant radiotherapy for primary liposarcoma (D, 2)	Should perform perioperative adjuvant radiotherapy (C, 2) It is not clear whether preoperative or postoperative irradiation is better for adjuvant radiotherapy (C, −)
CQ6	Is particle therapy recommended for primary retroperitoneal sarcoma?	Heavy ion radiotherapy should be performed for primary retroperitoneal sarcoma cases with difficulty of resection (D, 2)	Particle therapy should be performed for difficult‐to‐resect malignant soft tissue tumors (D, 2)
3. Treatment of unresectable retroperitoneal sarcoma			
CQ9	Is debulking surgery recommended for unresectable retroperitoneal sarcoma?	No clear recommendations can be made at this time for debulking surgery for unresectable retroperitoneal sarcoma (D, −)	Should resect the primary tumor in patients with malignant soft tissue tumors with distant metastases (D, 2) Should resect metastatic lesions in patients with malignant soft tissue tumors with distant metastases (D, 2)
CQ10	Is chemotherapy recommended for advanced retroperitoneal sarcoma?	No clear recommendations can be made at this time for drug therapy for advanced retroperitoneal sarcoma (D, −)	Should perform chemotherapy for metastatic malignant soft tissue tumor (C, 2) Doxorubicin monotherapy is recommended as first‐line treatment (B, 1)
CQ11	Is radiotherapy recommended for unresectable retroperitoneal sarcoma?	No clear recommendations can be made at this time for radiotherapy for unresectable retroperitoneal sarcoma (D, 2)	Not mentioned

Abbreviations: CQ, clinical question; JOA, Japanese Orthopaedic Association.

^a^ Strength of evidence: A: strong; B: moderate; C: weak; D: very weak; degree of recommendation: 1: strong; 2: weak.

### 3.2 The diagnosis of RPS

#### 3.2.1 Symptoms

Symptoms of RPS include a palpable mass, abdominal distension, abdominal and/or back pain, gastrointestinal obstruction, and urinary tract obstruction. However, these symptoms do not appear until the tumor has grown to some extent, so tumors are often quite large when discovered. In Japan, RPS is most often found incidentally during health checkups.^20^

#### 3.2.2 Diagnostic imaging

Abdominopelvic contrast‐enhanced computed tomography (CT) is the most useful examination for estimating tumor progression and histological type and for planning biopsy or surgery. Chest CT is also useful for staging RPS. In the guidelines, the usefulness of magnetic resonance imaging (MRI) and 18F‐fluorodeoxyglucose positron emission tomography (FDG‐PET)/CT for diagnosing RPS is discussed in CQ2.^4^

Owing to its high contrast resolution, MRI is useful for detecting pelvic lesions or diagnosing neuropore invasion. MRI has also been reported to be useful for diagnosing liposarcoma.^21^ However, liposarcoma is the only lesion for which such utility has been described, and there are many other types of RPS, as mentioned in chapter II. The guidelines therefore state that evidence is insufficient at present to insist on the utility of MRI for the diagnosis of general RPS.^4^

It has been suggested that PET/CT contributes to the evaluation of tumor malignancy and the early diagnosis of recurrence and metastasis. PET/CT may be useful for detecting multiple lesions or distant metastases, but high‐definition CT has been reported to be more useful than PET/CT for detecting small lung metastases.^22^ The usefulness of PET/CT for the diagnosis of RPS thus remains controversial.

#### 3.2.3 The pathological diagnosis

There are many histological types of retroperitoneal tumors, and the degree of malignancy differs for each histological type. Therefore, in CQ1, a biopsy before starting treatment is recommended to help determine the treatment strategy and predict the prognosis.^4^ In the soft tissue tumor guidelines, the recommendation of the biopsy method is precisely described according to the tumor size (<2 or >5 cm).^3^ However, it is relatively rare for RPS to be found at such small sizes. Therefore, it seems inevitable that the suggestion for a biopsy would be unspecific.

A biopsy is not always necessary when the histological type can be estimated from imaging findings alone (e.g., well‐differentiated liposarcoma) or when a biopsy is judged to be highly invasive or carry a high risk. There is some concern about the risk of tumor dissemination due to a needle biopsy, but one report described the risk as small.^23^ However, the impact of biopsy tract resection has not yet been confirmed.

RPSs are often large, and an accurate evaluation of the margin of the resected lesion is often difficult, so evaluation methods are not yet standardized.

### 3.3 Treatment of primary RPS

#### 3.3.1 Surgery

Surgery is the most effective treatment for RPS. In primary cases, resection should be performed with the aim of leaving no gross residual tumors. Although it is often difficult to achieve microscopically negative margins over the entire surgical margin (R0), resection without a grossly uncovered tumor, including the surrounding organs, is key to a good postoperative local control.^24^, ^25^, ^26^ Hence, CQ3 recommends R0 resection.^4^

However, the actual extent of resection should be determined by considering the location of the tumor, adjacent organs, and balance between functional preservation and radical resection. Of note, there is no specific description of the surgical techniques in the guidelines. Details of the actual surgical technique are described in an article from the European Organization for Research and Treatment of Cancer (EORTC)‐Soft Tissue and Bone Sarcoma Group (STBSG) and in an article from the Trans‐Atlantic RPS Working group.^2^, ^27^ Surgeons should therefore reference these articles when dealing with difficult‐to‐resect cases.

Wide resection for malignant cases has been suggested in the JOA clinical practice guidelines on the management of soft tissue tumors.^3^ This is because most malignant soft tissue tumors originate from the extremities, and wide resection of these tumors is easier than that of RPS.

Debulking surgery for unresectable RPS may provide symptomatic relief for patients debilitated by tumors. Several studies have reported that incomplete resection has a better survival time than an open biopsy; therefore, debulking surgery may improve the survival in select cases.^28^, ^29^, ^30^ However, incomplete resection or an open biopsy has been reported to be associated with a high rate of surgery‐related mortality (12%–17%).^19^, ^28^ Another study reported that surgery for patients with lower gastrointestinal obstruction was associated with complications in 60% of cases, with a mortality rate of 17%.^31^ Regarding the persistence of the effect of the operation, it was reported that approximately 70% of patients who underwent incomplete resection showed postoperative symptom improvement.^30^, ^31^ However, how long this improvement was maintained is unclear.

Well‐differentiated liposarcoma is a histologically low‐grade, slow‐growing tumor. Accordingly, debulking surgery is expected to show some efficacy in well‐differentiated liposarcomas. However, in other types of RPS, the duration of symptom relief is estimated to be short. Thus, no clear recommendation can be made regarding debulking surgery for unresectable RPS in CQ9.^4^

However, there is no description of debulking surgery in the JOA clinical practice guidelines for the management of soft tissue tumors. Most malignant soft tissue tumors originate from the extremities, so the idea of debulking surgery is not suitable for such cases. Instead of debulking surgery, resection of the main tumor or metastatic tumor for metastatic soft tissue tumors is suggested in the JOA guidelines.^3^

#### 3.3.2 Chemotherapy

The value of adjuvant chemotherapy for soft tissue sarcomas, including those of primary retroperitoneal origin, has not yet been established. Multiple randomized controlled trials of whole soft tissue sarcomas have been conducted; however, the results have been inconsistent. In a meta‐analysis reported in 2008, the doxorubicin–ifosfamide therapy group showed a better survival than the control group, with a hazard ratio (HR) of 0.56 (95% confidence interval [CI]: 0.36–0.85, *p* = 0.01).^32^ Conversely, in a pooled analysis of two STBSG–EORTC phase III clinical trials reported in 2014, the adjuvant chemotherapy group showed a better recurrence‐free survival than the control group, with an HR of 0.74 (95% CI; 0.60–0.92, *p* = 0.0056); however, adjuvant chemotherapy did not improve the overall survival.^33^

There are only retrospective reports of adjuvant chemotherapy for RPS. One is an analysis using data extracted from the National Cancer Database of America, and the other is a case series of consecutive cases from a single center.^34^, ^35^ Both studies reported that perioperative chemotherapy worsened the prognosis. Since these were retrospective studies, we cannot discount the possibility of existing selection bias, wherein adjuvant chemotherapy may have been administered to patients who were predicted to have a relatively poor prognosis. However, as no effective chemotherapy has been reported for RPS, there is no evidence to recommend perioperative chemotherapy. Thus, no clear recommendations can be made for adjuvant chemotherapy in CQ4.^4^

For unresectable advanced or recurrent soft tissue sarcoma, doxorubicin monotherapy is recommended as the first‐line therapy in the JOA clinical practice guidelines on the management of soft tissue tumors, 3rd edition.^3^ However, the JOA guidelines are mainly intended for orthopaedic malignant soft tissue sarcomas, which occur in the extremities and trunk. To date, no prospective clinical trial for chemotherapy in advanced or recurrent RPS has been conducted. Some retrospective studies have reported that doxorubicin‐based chemotherapy was effective in reducing tumors to a certain extent.^36^, ^37^, ^38^ Hence, doxorubicin‐based drugs may be effective as first‐line treatments for unresectable cases. Based on the results of several reports on sarcoma (overall), pazopanib, trabectedin, and eribulin are currently covered by Japanese insurance as second‐line treatments for soft tissue sarcoma.^39^, ^40^, ^41^ However, none of the reports demonstrating the efficacy of these agents have included subgroup analyses of RPS. Given the above, CQ10 states that the advantages and disadvantages of chemotherapy for unresectable RPS cannot be fully evaluated.^4^

#### 3.3.3 Radiotherapy (including particle therapy)

In surgical treatment of sarcomas, it is important to secure appropriate resection margins. However, RPSs are often located adjacent to important organs, making it difficult to secure appropriate resection margins. Radiotherapy is therefore sometimes performed as adjuvant therapy.

The results of the EORTC‐62092 (STRASS) trial, the only multicenter randomized controlled trial of neoadjuvant radiotherapy for RPS, were published in 2020.^42^ In this trial, the abdominal recurrence‐free survival (ARFS) was set as the primary endpoint, and the superiority of neoadjuvant radiotherapy for RPS over surgery alone was verified. However, no significant difference was noted between the two groups (HR: 1.01 [95% CI: 0.71–1.44], *p* = 0.95). A subgroup analysis of this trial showed that, in liposarcoma, which accounted for 74% of the enrolled patients, the 3‐year ARFS of the neoadjuvant radiotherapy group was better than that of the surgery‐alone group (HR: 0.64 [95% CI: 0.40–1.01]). Based on the results of this trial, the efficacy of neoadjuvant radiotherapy for promoting the ARFS was ruled out; however, there are still no data on the overall survival. Based on the above, it was stated that a clear recommendation of adjuvant radiotherapy for primary RPS could not be given at this time in CQ5‐i, and the delivery of adjuvant radiotherapy for primary retroperitoneal liposarcoma was suggested in CQ5‐ii.^4^

Proton therapy and heavy‐ion radiation therapy have been used in clinical practice in Japan. Compared with conventional radiotherapy, particle beam therapy has a stronger cell‐killing effect and can focus a particle beam on the tumor, reducing radiation damage to normal tissue around the radiation field, so stronger anti‐tumor effects can be expected.

There has only been one study on the efficacy of heavy ion beam therapy for primary difficult‐to‐resect or recurrent cases.^43^ The 2‐ and 5‐year overall survival rates for difficult‐to‐resect RPS treated with heavy ion therapy alone were found to be 75% and 50%, respectively, and the 2‐ and 5‐year local recurrence‐free survival rates were 77% and 69%, respectively. No grade ≥3 complications were observed after treatment with heavy ion irradiation alone. Although only one paper on this topic was reviewed, it is suggested that heavy‐ion radiotherapy for difficult‐to‐resect RPS is an effective treatment with relatively few side effects. Thus, CQ6 suggested that heavy ion radiotherapy be applied for difficult‐to‐resect cases.^4^

There have been only a few reports regarding proton beam therapy as neoadjuvant therapy, and no studies have discussed the effectiveness of proton beam therapy alone.^44^, ^45^ Therefore, the current guidelines have postponed making the decision of the recommendation for proton beam therapy.

In the JOA clinical practice guidelines on the management of soft tissue tumors, chemotherapy and radiotherapy are listed as clinical strategies for unresectable soft tissue malignant tumors.^3^ However, in the JOA guidelines, the rationale for this is not specified, and it is only described as having been performed in daily clinical practice. In clinical practice, there are no options other than radiotherapy or chemotherapy as first‐line treatment for unresectable local lesions. Radiotherapy has been applied to unresectable lesions based on the anti‐tumor effect suggested by evidence from comparative studies of surgery alone versus surgery with adjuvant radiotherapy. There are several retrospective reports on the results of radiation therapy for unresectable cases, but there are no data comparing such cases with patients managed without radiation therapy.^46^, ^47^ The usefulness of radiation therapy cannot be clearly determined. Thus, CQ11 made no clear recommendations concerning the application of radiotherapy for unresectable RPS.^4^

## 4 CONCLUSION

This review introduces the clinical problems encountered in the diagnosis and treatment of RPS and assesses these problems along with the CQs in the Japanese clinical practice guidelines for RPS. Although chemotherapy and radiotherapy are expected to have therapeutic effects, the level of evidence supporting these treatments is limited. Complete tumor resection is currently the first and only option for primary RPS. However, for other tumors, the demand for multidisciplinary treatment of RPS is increasing. These guidelines will be a milestone in clinical practice in relation to RPS in the future, and the accumulation of further evidence based on the CQs that have already been raised is expected.

## FUNDING INFORMATION

There was no external funding for this study.

## ETHICS STATEMENT

Approval of the research protocol: N/A.

Informed Consent: N/A.

Registry and the Registration No. of the study/trial: N/A.

Animal Studies: N/A.

## CONFLICT OF INTEREST STATEMENT

The authors declare no conflicts of interest for this article.
